# Investigating T-cell subpopulation-associated cytokine imbalance in vitiligo pathogenesis and the role of traditional Chinese medicine: A review

**DOI:** 10.1097/MD.0000000000047997

**Published:** 2026-03-20

**Authors:** Shidi Zhang, Ruoyang Zhao, Wenchao Yu, Huige Wang, Yuanhong Wang

**Affiliations:** a Heilongjiang University of Chinese Medicine, Harbin, China.

**Keywords:** Chinese medicine formula, cytokines, immune response, T cells, traditional Chinese medicine, vitiligo

## Abstract

Vitiligo, known as “Bai Bo Feng” in traditional Chinese medicine (TCM), is a prevalent skin disorder characterized by the loss of pigment, manifesting as white patches on the skin and the mucous membranes. While the diagnosis is straightforward, effective treatment remains elusive due to frequent recurrences. This study investigated the therapeutic mechanisms of TCM for vitiligo. A literature review was conducted to evaluate the association between T cell-related cytokines and vitiligo, and to examine the active ingredients in TCM and traditional Chinese medicine formulas (TCMFs) that show promise in the treatment of vitiligo. Various Chinese herbal extracts have been identified, including *Salvia miltiorrhiza*, *Artemisia annua*, and *Tribulus terrestris*, which exhibit effects on T-cell modulation. Traditional Chinese medicine formulas have also demonstrated clinical efficacy, showing reduction in cytokine secretion, thus mitigating autoimmune responses against melanocytes. This review provides evidence for the role of TCM and TCMFs in managing vitiligo through multi-target and multi-pathway interventions. Traditional Chinese medicine could present a promising avenue for treatment by affecting T-cell and cytokine profiles. Further research is essential to elucidate the complex mechanisms of action of TCM and TCMFs, which could lead to more effective treatment strategies for vitiligo.

## 1. Introduction

Vitiligo is an autoimmune dermatological disorder defined by the presence of well-demarcated hypopigmented patches, resulting from the selective loss of melanocytes. While readily identifiable, the condition presents significant challenges in achieving effective treatment and preventing recurrence.^[1]^ Its recurrent and refractory nature substantially impacts patients’ quality of life, with rising incidence rates linked to accelerated modern lifestyles and increasing societal pressures.^[2-4]^

Recent studies have confirmed that T cells and their associated cytokines play central roles in the pathogenesis of vitiligo.^[5,6]^ Chinese medicine is gradually gaining public and academic attention in China, especially in treating vitiligo.^[7,8]^ Herbal extracts like *Salvia miltiorrhiza*, *Artemisia annua*, and *Tripterygium wilfordii* show therapeutic potential for vitiligo.^[9,10]^ These herbal extracts modulate T-cell proliferation and regulate cytokine expression, thereby influencing the immune response in vitiligo. Some validated herbal formulas also target T cell molecules in vitiligo treatment. These formulas inhibit T-cell over-activation, regulate cytokine secretion, and attenuate autoimmune responses against melanocytes through a multi-target and multi-pathway strategy. A TCMF has been shown to reduce CXC chemokine ligand (CXCL) 10 content and cytotoxic T cells (CD8+) recruitment.^[11]^ A study^[12]^ proved that TCMF can reduce pro-inflammatory factors. A Chinese medicine formula^[13]^ can increase melanin content and may function through the PD-1/PD-L1 signaling pathway. A Chinese herbal formula,^[14]^ in combination with phototherapy, can elevate the expression of antioxidant and anti-inflammatory genes and reduce the ratio of CD3+ and CD8+ T cells. These herbal formulas are complex but offer a wide range of therapeutic options, and their mechanisms of action involve multiple dimensions. This review focuses on deepening the understanding of their therapeutic mechanisms and aims to provide scientific support for developing more effective therapeutic strategies.

## 2. Mechanism of vitiligo

### 2.1. Genetic factors

Recent advances have been made in the field of vitiligo. Vitiligo’s pathogenesis is clearer now, involving genetics, oxidative stress, and immune responses. Studies have shown that vitiligo tends to cluster in families, indicating a strong genetic component. Approximately 8% of patients have at least 1 affected relative, suggesting that genetic factors have a significant influence on the development of the disease.^[15]^ Studies of European descent have determined multiple susceptibility loci for vitiligo.^[16]^ In particular, studies on Han Chinese individuals with vitiligo have pointed to the granzyme B gene as a critical susceptibility locus.^[17]^ Furthermore, several studies have found that most of the susceptibility loci for vitiligo are associated with the genetic background of autoimmune diseases, further emphasizing the important role of the immune system in the pathogenesis of vitiligo.^[16]^

### 2.2. Oxidative stress

Oxidative stress has long been a popular topic in vitiligo research. Excessive accumulation of reactive oxygen species (ROS) occurs when certain specific environmental stimuli occur, and the average amount of ROS in the body mistakenly binds to phenolic compounds. Melanin production and mitochondrial metabolism are abnormal, and the expression of related genes is suppressed.^[18]^ Reactive oxygen species accumulation directly leads to melanocyte apoptosis,^[19]^ further damaging melanocyte function or triggering cell death through excessive cytokine production.^[20]^ Several recent studies have shown that oxidative stress may exert a vital impact on the pathogenesis of vitiligo by inducing the production of neoantigens in the relationship between oxidative stress and vitiligo. This theory has opened new avenues for subsequent research on vitiligo.^[21]^

### 2.3. Immune response

T cells play a vital role in immune responses, and abnormalities in their development and function are closely linked to the onset and recurrence of vitiligo.^[22-24]^ CD4+ and CD8+ T cells originate in the thymus as naïve cells.^[25-28]^ Upon antigen exposure, CD4+ T cells become activated, proliferate, and differentiate into various subsets (such as Th1, Th2, Th17, and Tfh) under the influence of specific cytokines, each performing distinct immune functions.^[29-31]^ CD8+ T cells, after recognizing antigens presented by antigen-presenting cells through their T cell receptors (TCRs),^[32-35]^ differentiate into either short-lived effector cells or long-lived memory cells in response to co-stimulatory signals and cytokines.^[36]^ Disruptions in T cell differentiation, activation, or related molecular signaling can lead to immune imbalances, contributing to diseases like vitiligo. The memory characteristics of T cells are crucial to the disease’s propensity for relapse and recurrence.^[37-39]^ Still, the specific role of T cells in the pathogenesis of vitiligo requires further study. In particular, the role of the ratio of Th17 and Treg cells in vitiligo, which is crucial in the immune regulation of inflammatory diseases, may provide a reference for vitiligo research by studying the significance of this ratio in lung diseases and inflammatory bowel diseases to promote the comprehensive understanding and application of the immune mechanism of vitiligo.^[40,41]^

## 3. Relationship between T cell subsets and vitiligo

Vitiligo is an autoimmune disorder primarily driven by T cell-mediated immune responses. Cytokines released by activated T cells contribute to melanocyte damage by inducing oxidative stress and disrupting intercellular adhesion. These cytokines further stimulate keratinocytes to secrete chemokines, which in turn recruit cytotoxic CD8+ T cells to the affected skin regions. Together, these processes lead to progressive melanocyte loss and depigmentation.

### 3.1. Cytokines and vitiligo

Cytokines are crucial in the pathogenesis of vitiligo.^[42]^ They mainly regulate immune responses and immune cell differentiation, and mediate inflammatory responses.^[43]^ Interferon-gamma (IFN-γ) is an important immunomodulatory cytokine produced mainly by activated T-cells and natural killer cells. It has multiple biological functions, including the activation of macrophages, enhancement of antigen presentation, promotion of cytotoxic responses, and regulation of immune responses.^[44]^ In autoimmune diseases such as vitiligo, abnormal expression of IFN-γ is closely related to melanocyte damage and can contribute to the onset of the disease.^[45]^ Recent studies over the past decade have shown that abnormal expression of IFN-γ contributes to the development of vitiligo through 3 primary pathways. The first pathway involves the disruption of E-cadherin, where elevated levels of interferon-gamma (IFN-γ) and tumor necrosis factor-alpha (TNF-α) promote its cleavage, resulting in weakened intercellular adhesion among melanocytes and their subsequent detachment.^[46]^ Concurrently, abnormal expression of IFN-γ induces keratinocytes to release proteins that further disrupt E-cadherin, causing melanocyte loss.^[47]^ The second one activates the JAK-STAT (Janus kinase-signal transducer and activator of transcription) signaling pathway, leading to the transcription of genes encoding relevant chemokines and cytokines, thereby facilitating the onset of vitiligo.^[48]^ The third pathway involves increased oxidative stress. Abnormal IFN-γ expression elevates oxidative stress levels, resulting in the accumulation of intracellular ROS and ultimately diminishing melanin production, which is a significant mechanism in the pathogenesis of vitiligo.^[49]^ Tumor necrosis factor-alpha is an inflammatory factor that may cause damage and death of melanocytes during vitiligo development through multiple mechanisms.^[50]^ Tumor necrosis factor-alpha is mainly produced by the Th1 and Th17 subsets of CD4+ T cells. In the skin, TNF-α is mainly secreted by keratinocytes, which can affect the expression of melanin-synthesizing enzymes by downregulating the functions of related transcription factors and reducing the expression of melanin receptors.^[51]^ TNF-α binds to the tumor necrosis factor receptor (TNFR) on the membrane of specific target cells, activating intracellular signal transduction pathways and triggering melanocyte apoptosis. Research has demonstrated that abnormal levels of TNF-α expression may activate the TNF-TNFR-mediated apoptosis signaling pathway, leading to melanocyte apoptosis. This mechanism is crucial in the development of vitiligo.^[52,53]^ Interleukin(IL)-17 is a cytokine primarily involved in regulating immune responses and inflammatory processes, recruiting neutrophils, and modulating the functions of immune cells.^[54]^ Some studies have indicated that elevated serum levels of IL-17 in patients with vitiligo are associated with disease activity, extent, and severity.^[55]^ Research has shown that oxidative stress may result in a reduction in the number of melanocytes and the abnormal expression of IL-17-related gene transcription factors. In addition to inhibiting melanocyte production, IL-17 may also cause mitochondrial dysfunction and promote autophagy in melanocytes.^[56]^

### 3.2. The relationship between chemokines and vitiligo

Chemokines may contribute to the development of vitiligo by guiding the migration and localization of immune cells and affecting the survival and migration of pigment cells.^[57]^ Under the stimulation of IFN-γ and IFN-α, keratinocytes release 2 chemokines, CXCL9 and CXCL10, which are responsible for recruiting CD8+ T cells to the skin, thereby triggering vitiligo.^[58]^ Certain studies have shown that the level of CXCL9 expression in the skin of individuals with vitiligo is significantly elevated, and this increase is strongly associated with the severity of the condition.^[59]^ A meta-analysis of CXCL10 showed that CXCL10 production in vitiligo patients is usually elevated compared to that in healthy controls.^[60]^ Furthermore, research has indicated that serum levels of CXCL10 and CXCL12 are markedly elevated in individuals with active vitiligo compared to those with stable disease, suggesting that these chemokines may be used to predict the activity status of vitiligo.^[61]^

CXCL16 is generated by keratinocytes in response to stimulation by reactive ROS and unfolded protein response.^[62]^ In the skin of vitiligo patients, the mRNA level of CXCL16 is associated with oxidative stress. In addition, CXCL16 regulates the migration of CD8+ T cells to vitiligo-damaged skin via its interaction with the CXC chemokine receptor (CXCR) 6 receptor. These findings suggest that the CXCL16–CXCR6 signaling axis plays a pivotal role in the immune mechanisms underlying vitiligo. According to Speeckaert et al, CXCL12 and CC chemokine ligand (CCL) 5, derived from melanocytes in vitiligo, play an initial role in the recruitment of immune cells and may also be involved in initiating and maintaining specific immune responses. CXCL12 and CCL5 serve as critical roles in the activation of melanocyte-specific immunity. Melanocyte transplants expressing CXCL12 and CCL5 can recruit T-cells and induce vitiligo lesions.^[63]^

CXCR3B is a unique subtype specifically expressed in human melanocytes.^[64]^ Current research has found that IFN-γ regulates this process and can be activated by CXCL10, triggering the apoptosis pathway. It is important to highlight that this critical mechanism has not been uncovered in traditional mouse model studies, because mouse melanocytes lack CXCR3B expression. Therefore, in-depth research on CXCR3B provides a new exploration route and research direction for treating skin pigmentation deficiency diseases, such as vitiligo.^[65]^

## 4. Intervention of traditional Chinese medicine

The application of TCM and TCMFs in the treatment of vitiligo has received increasing attention in recent years. Emerging evidence suggests that active ingredients within TCM and TCMFs may exert therapeutic effects by modulating T cell activity, primarily through the regulation of cytokine secretion and intracellular signaling pathways.

### 4.1. Active ingredients of TCM

Currently, TCM is gradually gaining widespread public attention in China, and its efficacy in disease treatment is increasingly attracting academic interest. Pharmacological research has identified a variety of Chinese herbal extracts and active ingredients that have shown potential efficacy in vitiligo treatment, including *S miltiorrhiza*, *A annua* L., *Tribulus terrestris* L., *Reseda odorata* L., Baical skullcap root, Licorice, *Paeonia lactiflora*, and *T wilfordii*. Herbal extracts can inhibit T cell proliferation and regulate cytokines, offering a practical approach to treating vitiligo.^[66-71]^ The active ingredients in Salvia and Artemisia mainly affect the expression levels of T cells. Mechanistically, *S miltiorrhiza* extracts, particularly tanshinones, have been shown to interfere with the TCR/CD3 complex signaling cascade, leading to reduced activation of proximal tyrosine kinases Lck and Fyn. This attenuation downstream suppresses the nuclear translocation of transcription factors like nuclear factor of activated T cells and nuclear factor kappa B (NF-κB), ultimately resulting in decreased transcription of genes encoding IFN-γ and perforin, thereby curbing CD8+ T cell cytotoxicity and melanocyte apoptosis.^[66]^ Artesunate reduces the number and ratio of CD4+ T cells by inhibiting the phosphorylation of JAK2 and STAT3 and interfering with the JAK/STAT signaling of IL-6 and IL-21.^[67]^ The active ingredients of Baical skullcap root, *R odorata* L., Licorice, and *P lactiflora*, namely baicalin, luteolin, glycyrrhizic acid, and paeoniflorin, respectively, can effectively intervene in T cell recruitment by reducing or inhibiting the serum levels of chemokines and cytokines. Luteolin functions by directly inhibiting IκB kinase (IKK) activity, which prevents IκBα degradation and sequesters NF-κB p65 subunits in the cytoplasm. This blockade of NF-κB signaling pathway leads to diminished transcriptional activation of the IL-8 gene and reduced IL-8 protein secretion, impairing T cell chemotaxis.^[68]^ In a study on the mechanism of action of baicalin in a mouse model of vitiligo, baicalin was found to be pivotal in the progression of vitiligo by effectively reducing the chemokines CXCL9, CXCL10, and CXCR3, reducing the recruitment of CD4+ T cells and CD8+ T cells, reducing the area and incidence of pigment loss in vitiligo mice, and elevating the number of melanocytes in the epidermis of depigmented skin. Glycyrrhizinic acid, the main component of licorice, appears to exert its effects partly by upregulating microphthalmia-associated transcription factor gene expression, which enhances tyrosinase activity and melanogenesis. Concurrently, it interferes with the binding of IFN-γ to its receptor through competitive inhibition, reducing downstream JAK-STAT signaling and CXCL10 gene transcription.^[69]^ Paeoniflorin exerts multifaceted effects. It promotes the proliferation of melanocytes (PIG3V cells) by activating the NF-κB signaling pathway. Conversely, under oxidative stress induced by H_2_O_2_, paeoniflorin significantly suppresses the expression of the inflammatory mediator GPR17 and inhibits the phosphorylation of the key NF-κB pathway component p65. This leads to reduced release of the pro-inflammatory cytokines IL-1β and IL-6, thereby mitigating GPR17-mediated inhibition of melanocyte proliferation, suppressing CD8+ T cell migration, and lowering inflammation levels in melanocytes.^[70]^ Paeoniflorin may also modulate the NF-κB pathway through allosteric regulation, preventing overactivation of the inflammatory response. One of the active ingredients in *T wilfordii* is demethylzeylasteral (T-96). Research has demonstrated that T-96 can significantly inhibit the proliferation of CD8+ T cells obtained from individuals with vitiligo, reduce the expression of CD69, and reduce the levels of IFN-γ, granzyme B, and perforin (PRF). In addition, T-96 can interact with Janus kinase 2 (JAK2) in IFN-γ, thereby inhibiting JAK2 activation and reducing the total protein and phosphorylated protein levels of signal transducer and activator of transcription 1 (STAT1). This effect further reduces the synthesis and release of the chemokines CXCL9 and CXCL10, thereby exhibiting a positive therapeutic effect on vitiligo.^[71]^

### 4.2. Intervention of TCMF

**Figure 1.**
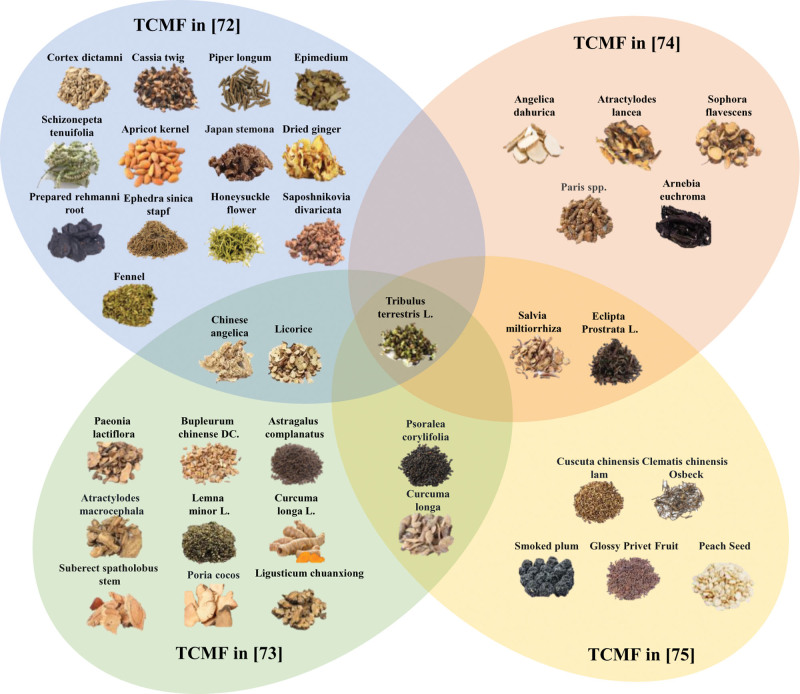
Composition of traditional Chinese medicine formulas for the treatment of vitiligo. A Venn diagram format is used to represent 4 different TCMFs. Each circle corresponds to 1 formula, and overlapping areas indicate shared herbal ingredients among them. Despite differences in overall composition, certain core herbs are consistently included across the formulas to achieve essential therapeutic effects. TGMF = traditional Chinese medicine formula.

Several clinically proven TCMFs (Fig. 1 and Table 1) have been shown to have significant effects in treating vitiligo, especially in terms of their unique impact on the intervention of T-cell-related molecules. In Figure 1, a Venn diagram format is used to represent 4 different TCMFs. Each circle corresponds to 1 formula, and overlapping areas indicate shared herbal ingredients among them. This presentation highlights that, despite differences in overall composition, certain core herbs are consistently included across the formulas to achieve essential therapeutic effects. These prescriptions reduce the attack of autoimmune responses on melanocytes by inhibiting the overactivation of T cells, regulating the secretion of cytokines, and affecting the interaction of T cells with other immune cells. These multi-pathway strategies offer more treatment options and a basis for exploring vitiligo’s pathogenesis and new treatments.

**Table 1:** **Summary of the effects of traditional Chinese medicine on CD4+, CD8**+ **T cells and related cytokines**.

Typology	Name	Intervention level*	Regulatory role†	References
Active ingredients of traditional Chinese medicine	*Salvia miltiorrhiza*	Cellular expression	Inhibition of active proliferation of CD8+ T cells	^[6^ ^5^^]^
	Artesunate	Cellular expression	Affects the JAK/STAT signaling pathway, reducing the number of CD4+ T cells.	^[6^ ^6^^]^
	Rhinocerosin	Secretory expression of related molecules	Inhibition of the release of the key in flammatory chemokine IL-8	^[6^ ^7^^]^
	Liquorice acid	Secretory expression of related molecules	Increases tyrosinase activity and inhibits CXCL10 expression	^[6^ ^8^^]^
	Baicalin	Cellular expression and associated molecular secretion	Reduced expression of CXCL9, CXCL10, CXCR3 chemokines, reduced recruitment of CD4+ T cells and CD8+ T cells	^[^^69^^]^
	Paeoniflorin	Cellular expression and associated molecular secretion	Affects NF-κB signaling pathway transduction, promotes PIG3V cell proliferation, decreases IL-1β, IL-6 release inhibits CD8+ T cell migration	^[^^70^^]^
	Demethylzelantanol	Cellular expression and associated molecular secretion	Inhibits proliferation of CD8+ T cells, reduces membrane expression of CD69, and decreases levels of IFN-γ, granzyme B, and perforin. Reduces the production and secretion of CXCL9 and CXCL10	^[^^71^^]^
Traditional Chinese medicine formulas	Formula in^[72^^]^	Secretory expression of related molecules	Reduced levels of TNF-α, IL-33, IL-6	^[^^72^^]^
	Formula in^[73]^	Cellular expression	Activation of the PD-1/PD-L1 signaling pathway in skin lesions inhibits the T-cell-mediated autoimmune response against melanocytes	^[^^73^^]^
	Formula in^[74]^	Cellular expression and associated molecular secretion	Decreased CXCL10 levels and reduced CD8+ T cell recruitment	^[^^74^^]^
	Formula in^[75]^	Cellular expression and associated molecular secretion	Up-regulation of Nrf2 and p62 mRNA expression, promotion of HO-1 expression, and reduction of the ratio of CD3+ CD8+ T cells in peripheral blood	^[7^ ^5^^]^

CCL = CC chemokine ligand, CD3 = cluster of differentiation 3, CD4+ = follicular helper, CD8+ = cytotoxic, CXCL = CXC chemokine ligand, CXCR = CXC chemokine receptor, IFN-γ = interferon-gamma, IL-17 = interleukin-17, JAK-STAT = Janus kinase-signal transducer and activator of transcription, NF-κB = nuclear factor kappa B, TNF-α = tumor necrosis factor-alpha.

* Intervention level describes the level at which the intervention occurs. This can include cellular expression, which refers to the modulation of immune cell activity at the cellular level, or secretory expression of related molecules, which refers to the effect of the compound on the secretion of cytokines, chemokines, or other signaling molecules that influence immune responses and cell recruitment.

† Regulatory role describes how the compound regulates immune responses, particularly in terms of modulating T cell activity, cytokine secretion, or chemokine production.

Wang et al discovered that after treatment with a TCMF made from *Ephedra sinica* stapf, Cassia Twig, *Schizonepeta tenuifolia*, *Saposhnikovia divaricata*, Epimedium, Apricot kernel, Japan stemona, Prepared Rehmannia, Fennel, Dried Ginger, *Piper longum*, *T terrestris* L., *Dictamnus dasycarpus*, Honeysuckle flower, *Chinese Angelica*, and Licorice, among others. It was found that after treatment, this TCMF can effectively reduce the content of the chemokine CXCL10 in the serum of patients and reduce the chemotactic recruitment of CD8+ T cells to participate in the process of melanocyte damage. Its central immunomodulatory mechanism involves a significant reduction in serum levels of the chemokine CXCL10. As a key signaling molecule, CXCL10 specifically recruits activated CD8+ T cells, particularly those expressing CXCR3, to sites of inflammation. By inhibiting the production or release of CXCL10, this treatment reduces the recruitment and cytotoxic activity of these T cells toward melanocytes.^[72]^ In their study, Li et al investigated the effects of a TCMF made from *Bupleurum chinense*, *Angelica sinensis*, *T terrestris*, *Psoralea corylifolia*, *Taraxacum mongolicum*, Ligusticum chuanxiong, *Spatholobus suberectus*, *P lactiflora*, *Curcuma longa*, *Spirodela polyrrhiza*, *Poria cocos*, *Atractylodes macrocephala*, *Curcuma longa*, and *Glycyrrhiza uralensis*, in treating patients with stable-stage vitiligo. They found that TCMF effectively reduced scores on the Self-Rating Anxiety Scale (SAS) and Self-Rating Depression Scale (SDS), and significantly decreased serum levels of TNF-α, IL-33, and IL-6, thereby achieving therapeutic effects for vitiligo.^[73]^ Wu et al found that a TCMF with a formula (*Angelica dahurica*, *T terrestris* L., *Atractylodes lancea*, *Sophora flavescens*, Paris spp., *Arnebia euchroma*, *S miltiorrhiza*, and *Eclipta prostrata* L.) can increase the melanin content in the skin of mice in a vitiligo model. The potential mechanism may lie in activating the PD-1/PD-L1 signaling pathway in lesional tissue and suppressing T cell-mediated autoimmune responses against melanocytes. These findings provide a new perspective on the treatment of vitiligo and highlight the potential value of TCM in regulating immune responses. This formula was shown to increase skin melanin content in a vitiligo mouse model. Its core underlying mechanism involves activating the PD-1/PD-L1 signaling pathway within the lesional tissue. PD-1 (programmed death-1) is primarily expressed on activated T cells, while its ligand PD-L1 is expressed by keratinocytes. Binding of PD-1 to PD-L1 delivers an inhibitory signal to the T cell, inducing functional exhaustion, suppressing proliferation, reducing cytokine production, and diminishing cytotoxic activity. Consequently, activating this pathway inhibits the attack by effector T cells on melanocytes.^[74]^ Zhang et al found that the combined application of the TCMF (which contains the ingredients Smoked plum, Glossy privet fruit, Clematis chinensis osbeck, *Cuscuta chinensis* lam, *C longa*, *P corylifolia*, *T terrestris* L., *E prostrata* L., *S miltiorrhiza*, and Peach seed) and 308 excimer light therapy showed significant efficacy in the treatment of progressive vitiligo. This treatment regimen can effectively enhance the expression of Nrf2 and p62 mRNA, promoting the expression of HO-1 downstream, thereby achieving antioxidant, anti-inflammatory, and immunomodulatory functions. In addition, this combination therapy significantly reduced the proportion of CD3+ CD8+ T cells in peripheral blood, and its comprehensive efficacy was superior to that of phototherapy alone. This finding provides a new strategy for treating vitiligo and highlights the potential advantages of combined Chinese and Western medical treatments in clinical applications.^[75]^

In the therapeutic strategy for vitiligo, conventional treatment primarily focuses on suppressing the immune response that damages melanocytes or promoting their proliferation and recovery. Specific methods include the use of topical agents such as corticosteroids, calcineurin inhibitors, and Janus kinase (JAK) inhibitors to modulate local immunity and reduce melanocyte destruction^[76]^; additionally, narrowband ultraviolet B (NB-UVB) phototherapy stimulates melanocyte proliferation and migration.^[77]^ For patients in the stable phase, surgical interventions like autologous epidermal transplantation or melanocyte transplantation may also be considered.^[78]^ In contrast, TCM adopts a more holistic approach, emphasizing syndrome differentiation and individualized treatment. Traditional Chinese medicine focuses on regulating immune responses and restoring internal immune balance through herbal formulas tailored to the patient’s constitution, disease duration, and accompanying symptoms. This approach aims to improve organ system function and promote vitiligo resolution. Traditional Chinese medicine treatments tend to work gradually and are often more suitable for patients in the stable phase of vitiligo. The 4 TCM formulas discussed share several key herbs, including *T terrestris*, *S miltiorrhiza*, *A sinensis*, *P lactiflora*, *G uralensis*, and *C longa*, which play central roles in the therapeutic effects of these treatments for vitiligo. These herbs exhibit complementary immunomodulatory, anti-inflammatory, and antioxidant properties. Tribulus terrestris, for example, regulates immune cell activity and reduces the production of pro-inflammatory cytokines, thereby helping to reduce T cell-mediated attacks on melanocytes. *S miltiorrhiza* and *C longa* both act on the NF-κB and MAPK pathways, reducing inflammatory responses and protecting melanocytes from oxidative stress. *A sinensis* and *P lactiflora* contribute to this immune modulation by balancing cytokine levels and inhibiting Th1 and Th17 responses, which are critical in vitiligo’s pathogenesis. *G uralensis* helps regulate the JAK-STAT signaling pathway, further limiting the activation of cytotoxic T cells. Together, these herbs work synergistically to suppress inflammation, regulate immune responses, and promote melanocyte protection, making them key components in TCM formulations aimed at treating vitiligo.

The aforementioned TCMFs have shown unique advantages in the treatment of vitiligo. It is comprised of various Chinese herbal medicines, has a complex composition and diverse functions, and provides patients with multiple treatment options. The above analyses showed that TCMFs can interact with T cells and their related molecules to affect the course of the disease. However, the mode of action of TCMF is complex. It involves multiple processes, such as signal transduction, gene regulation, and molecular interactions, which are not yet fully understood. Nevertheless, most of the cited studies on TCM formulas are based on small-scale trials or animal models, and there is a lack of large-scale randomized controlled trials (RCTs). To address this gap, future research should consider the adoption of strategies like chemical fingerprinting or quality control markers for herbal formulations to standardize TCMF preparations. Future research should also explore the mechanism of action of TCMFs in vitiligo treatment, including signaling pathways, genes, and molecular interactions, to obtain a more thorough understanding of their therapeutic mechanisms and to provide a scientific basis for developing more effective and safer treatment strategies. Based on the above discussion, the modulatory effects of TCM and TCMFs on T cell and its related molecules are summarized (Table 1). Moreover, a detailed comparison between conventional treatments and TCM for vitiligo is as shown in Table 2. This comparison outlines the primary mechanisms, onset of action, side effects, and suitable patient subgroups for each treatment approach.^[79-82]^ To overcome the current limitations, future studies should prioritize large-scale, well-designed RCTs to validate the clinical efficacy and safety of TCMFs.

**Table 2:** **Comparison of TCM and conventional treatments for vitiligo**.

Feature	Conventional treatment	Traditional Chinese medicine (TCM)	References
Primary mechanism	• Suppress local and systemic immune overactivation (e.g., corticosteroids, calcineurin inhibitors, JAK inhibitors) • Promote melanocyte proliferation and migration (e.g., NB-UVB) • Replace melanocytes directly (surgical transplantation)	• Modulate T-cell activity (reduce CD8+ T-cell infiltration, rebalance Th17/Treg, inhibit cytokines such as IFN-γ, TNF-α, IL-17) • Reduce chemokines (e.g., CXCL9/CXCL10) and inhibit immune recruitment • Enhance antioxidative capacity via Nrf2/HO-1 pathway	^[79]^
Onset of action	• Generally rapid (weeks to a few months), depending on modality • Phototherapy shows visible repigmentation within 1–3 mo	• Gradual onset; therapeutic effect often requires months of continuous treatment • More suitable for long-term immune modulation	^[80]^
Common side effects	• Long-term corticosteroid use may lead to skin atrophy, pigment changes, osteoporosis, etc • Phototherapy can cause skin dryness, erythema, or phototoxic reactions	• Herbal formulas may cause mild gastrointestinal discomfort, elevated liver enzymes, etc • Some herbs may lead to allergic reactions or liver/kidney burden, requiring professional guidance for use	^[81]^
Suitable patient subgroups	• Patients with active or progressive vitiligo requiring rapid immune suppression • Suitable for both short-term control and induction of repigmentation	• Patients with stable or chronic vitiligo • Individuals intolerant to corticosteroids or seeking complementary and holistic approaches	^[82]^

CD8+ = cytotoxic, CXCL = CXC chemokine ligand, IFN-γ = interferon-gamma, IL-17 = interleukin-17, JAK = Janus kinase, NB-UVB = narrowband ultraviolet B, TCM = traditional Chinese medicine, TNF-α = tumor necrosis factor-alpha.

## 5. Conclusion

T cells and their associated cytokines play a pivotal role in the pathogenesis of vitiligo, primarily by mediating the destruction of melanocytes. Traditional Chinese medicine has shown promising immunomodulatory effects on these immune mechanisms, offering a potential therapeutic approach for vitiligo management. This review identifies specific TCM ingredients and formulations that modulate T cell activity and cytokine expression, suggesting their potential utility in treating vitiligo. However, in contrast to conventional therapies, such as topical corticosteroids, TCM presents certain limitations. Traditional Chinese medicine treatments generally have a gradual onset of action and often require extended periods of administration to achieve therapeutic effects, making them particularly suitable for patients in the stable phase of vitiligo. Further research is necessary to elucidate the specific immunomodulatory pathways by which TCM influences vitiligo. There is also potential for TCM to be used as an adjuvant therapy during the active phase of vitiligo, supporting the effects of conventional treatments or reducing their side effects. Future studies should focus on examining the direct effects of key TCM compounds on T cell subsets, such as Th1, Th17, and regulatory T cells. Additionally, well-designed experimental studies and large-scale clinical trials are essential to validate the clinical efficacy and safety of TCM-based therapies. Standardizing TCM formulations and treatment protocols will be crucial for enhancing their clinical applicability. Collectively, these efforts will contribute to the development of more effective, targeted strategies for vitiligo treatment.

## Author contributions

**Conceptualization:** Shidi Zhang.

**Funding acquisition:** Yuanhong Wang.

**Supervision:** Yuanhong Wang.

**Visualization:** Shidi Zhang, Ruoyang Zhao, Wenchao Yu, Huige Wang.

**Writing – original draft:** Shidi Zhang.

**Writing – review & editing:** Ruoyang Zhao, Wenchao Yu, Huige Wang.
